# Distinct 3-*O*-Sulfated Heparan Sulfate Modification Patterns Are Required for *kal-1*−Dependent Neurite Branching in a Context-Dependent Manner in *Caenorhabditis elegans*

**DOI:** 10.1534/g3.112.005199

**Published:** 2013-03-01

**Authors:** Eillen Tecle, Carlos A. Diaz-Balzac, Hannes E. Bülow

**Affiliations:** * Department of Genetics, Albert Einstein College of Medicine, Bronx, New York, 10461; † Dominick P. Purpura Department of Neuroscience, Albert Einstein College of Medicine, Bronx, New York, 10461

## Abstract

Heparan sulfate (HS) is an unbranched glycosaminoglycan exhibiting substantial molecular diversity due to multiple, nonuniformly introduced modifications, including sulfations, epimerization, and acetylation. HS modifications serve specific and instructive roles in neuronal development, leading to the hypothesis of a HS code that regulates nervous system patterning. Although the *in vivo* roles of many of the HS modifications have been investigated, very little is known about the function of HS 3-*O-*sulfation *in vivo*. By examining patterning of the *Caenorhabditis elegans* nervous system in loss of function mutants of the two 3-*O-*sulfotransferases, *hst-3.1* and *hst-3.2*, we found HS 3-*O*-sulfation to be largely dispensable for overall neural development. However, generation of stereotypical neurite branches in hermaphroditic-specific neurons required *hst-3.1*, *hst-3.2*, as well as an extracellular cell adhesion molecule encoded by *kal-1*, the homolog of Kallmann Syndrome associated gene 1/anosmin-1. In contrast, *kal-1*−dependent neurite branching in AIY neurons required catalytic activity of *hst-3.2* but not *hst-3.1*. The context-dependent requirement for *hst-3.2* and *hst-3.1* indicates that both enzymes generate distinct types of HS modification patterns in different cell types, which regulate *kal-1* to promote neurite branching. We conclude that HS 3-*O-*sulfation does not play a general role in establishing the HS code in *C. elegans* but rather plays a specialized role in a context-dependent manner to establish defined aspects of neuronal circuits.

During nervous system development, growing neurons have to interact with the extracellular environment to establish functional neuronal circuits. Parts of the extracellular environment are extracellular matrix components, such as heparan sulfate (HS) proteoglycans, which mediate cellular interactions during development (Bernfield *et al.* 1999; Ramirez and Rifkin 2003). HS are linear glycosaminoglycan polysaccharides with a substantial heterogeneity as a result of modifications, such as sulfations, epimerization, and acetylation (Lindahl and Li 2009). The HS chains are attached to conserved HS core proteins like the membrane-bound syndecans, glypicans, and the secreted perlecan, collagen XVIII and agrin (Bernfield *et al.* 1999). HS synthesis and modification occurs in the Golgi, where membrane-associated type-II HS modification enzymes act on disaccharide repeats of glucuronic acid and *N*-acetylglucosamine (Figure 1A), resulting in unbranched HS chains of 50−150 disaccharide repeats (Lindahl and Li 2009). During biosynthesis in the Golgi, HS modifications occur, including the partial deactelylation and sulfation of the glucuronic acid and *N*-acetylglucosamine residues by the enzyme *N*-deacetylase/*N*-sulfotransferase (Figure 1A). In addition to epimerization of select hexuronic acid residues by the glucuronic C-5 epimerase (Figure 1A), the introduction of sulfate groups to different positions of the sugars is achieved by highly selective 2-*O*, 6-*O* and 3-*O* HS sulfotransferases (Figure 1A). The HS modification enzymes do not act on every sugar, resulting in nonuniformly and nonrandomly modified regions along a single HS chain.

**Figure 1:**
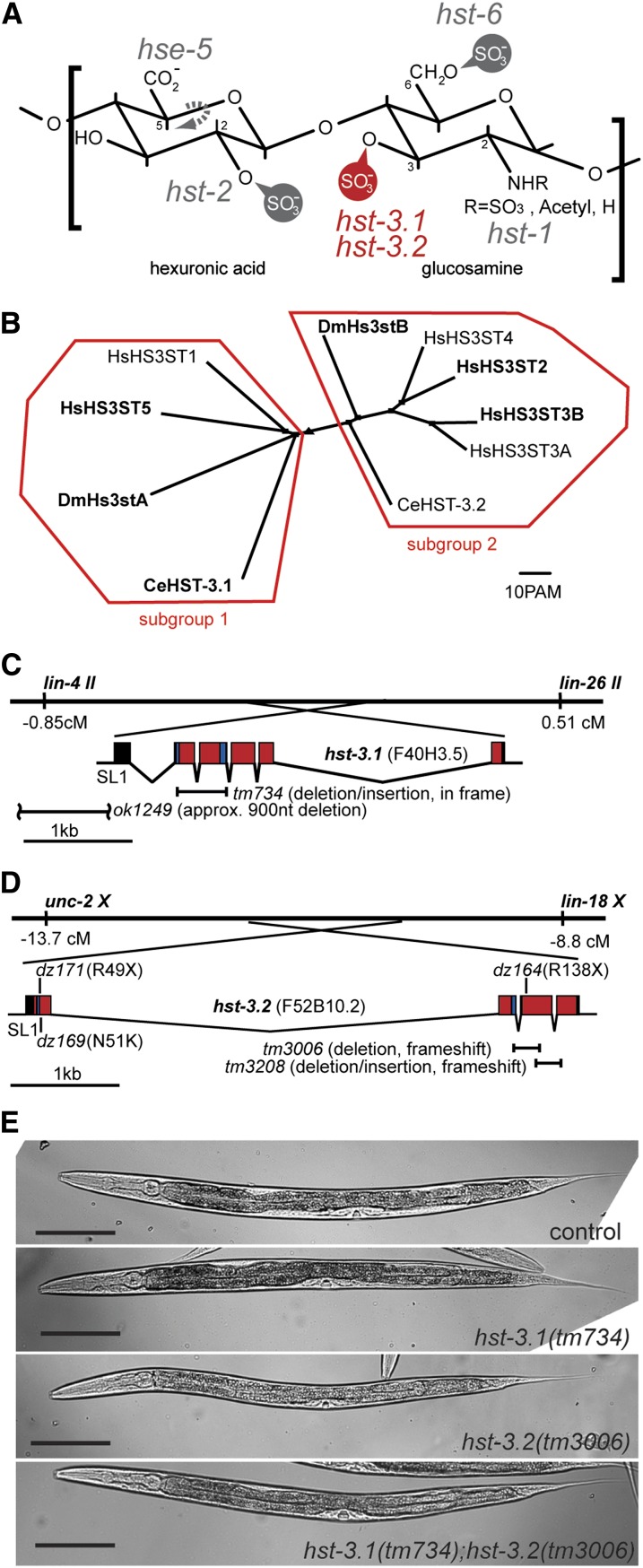
HS 3-*O*-sulfotransferases in *C. elegans*. (A) Characteristic HS disaccharide comprising a hexuronic acid (glucuronic acid or iduronic acid) and glucosamine. The *C. elegans* genes coding for HS modification enzymes are indicated next to the positions they modify: *hse-5*, HS C5 glucuronyl epimerase; *hst-2*, HS 2-*O*-sulfotransferase; *hst-6*, HS 6-*O*-sulfotransferases; *hst-1*, *N*-deacetylase/sulfotransferase; *hst-3.1* and *hst-3.2*, HS 3-*O*-sulfotransferases (in red). (B) Phylogenetic clustering of HS 3-*O*-sulfotransferases with ClustalW into two subgroups. Accession numbers: HsHS3ST1 (NP_005105), HsHS3ST5 (NP_705840), DmHs3stA (NP_788409), DmHs3stB (NP_573370), HsHS3ST4 (Q9Y661), HsHS3ST2 (NP_006034), HsHS3ST3B (NP_006032), HsHS3ST3A (NP_006033), CeHST-3.1 (CCD66155), and CeHST-3.2 (NP_001024698). Proteins in bold have a predicted transmembrane domain using a hidden Markov Model (Krogh *et al.* 2001) at http://www.cbs.dtu.dk/services/TMHMM/. There is no obvious correlation between subgroups and predicted transmembrane domains. (C) Gene structure of *hst-3.1* on chromosome II. Based on cDNA analyses, the *tm734* allele results in an in frame deletion of 88 amino acids and is a predicted strong loss of function allele (Figure 2). Based on PCR analyses and sequencing, the *ok1249* allele results in an approximate 0.9kb deletion of the promoter leaving at least 125 bp upstream of the *hst-3.1* transcription start site. The *hst-3.1* transcript is transpliced to SL1. In panels C and D, exons shaded in red encode the sulfotransferase domains and 5′- and 3′-PAPS binding sites are indicated in blue. (D) Gene structure of *hst-3.2* on chromosome X. The *hst-3.2* transcript is transpliced to SL1. The position of deletion and point mutant alleles are shown. Based on cDNA analyses, the *hst-3.2(tm3006)* allele results in a frameshift after 121 of 291 amino acids and a premature stop after two additional nonconserved amino acids (Figure 2B) whereas *hst-3.2(tm3208)* creates a frameshift after 175 of 291 amino acids with a premature stop after nine nonconserved amino acids (Figure 2B). This leaves a structural determinant of the enzyme partially intact, namely a loop that is predicted to partake in the formation of the groove in which the HS substrate binds (Figure 2, C−E) (Edavettal *et al.* 2004). Point mutant alleles were isolated by expanding a screen described previously (Bülow *et al.* 2002) and result in stop codons after 49 amino acids (*dz171*), 138 amino acids (*dz164*), or a N51K missense mutation (*dz169*) which introduces a positive charge in the cosubstrate binding site (Figure 2B). (E) DIC images of control, *hst-3.1*, *hst-3.2*, and double mutants. Both single and double mutants of *hst-3.1* and *hst-3.2* are viable, fertile and display no obvious morphological defects. Scale bar indicates 100 µm.

HS glycans serve developmental and physiological roles by functioning in multiple signaling pathways (reviewed in Bülow and Hobert 2006 and Bishop *et al.* 2007). Knockout studies of HS modification enzymes in vertebrates and invertebrates suggest that some of the functions of HS are mediated by the complex modification patterns of HS that act as protein binding sites (reviewed in Bülow and Hobert 2006 and Bishop *et al.* 2007). Genetic experiments in *Caenorhabditis elegans* suggest that HS can act instructively, possibly by directly modulating ligand/receptor interactions (Bülow *et al.* 2008). Alternatively, they may serve to immobilize secreted ligands, thereby aiding in the development of ligand gradients in the extracellular space (Lindahl and Li 2009).

The rarest and most enigmatic of all HS modifications has been the 3-*O*-sulfation of the glucosamine residue, which is estimated to contribute at most 0.5% of total sulfate in a HS chain (Shworak *et al.* 1994). Despite its rarity, seven genes are part of the vertebrate 3-*O*-sulfotransferase (HS3ST) gene family (reviewed in Lindahl and Li 2009), indicating that 3-*O-*sulfation may serve important functions. HS3STs fall into two subgroups (Liu *et al.* 1999; Cadwallader and Yost 2006): Members of subgroup 1 can form a HS modification pattern required for antithrombin binding to HS (Shworak *et al.* 1997; Shworak *et al.* 1999), and members of subgroup 2 can create a HS modification pattern that mediates herpes simplex virus-1 infection of Chinese hamster ovary cells in culture (Shukla *et al.* 1999). These studies have led to the general concept that HS 3-*O*-sulfation is critical for the generation of high-affinity binding sites (Lindahl and Li 2009). However, little is known about the *in vivo* roles of HS 3-*O*-sulfation. Genetic removal of HS3ST1 in mice, potentially the most ubiquitously expressed HS 3-*O*-sulfotransferase in vertebrates, results in intrauterine growth retardation and postnatal death depending on the genetic background but surprisingly no defects in blood coagulation due to defective thrombin neutralization (HajMohammadi *et al.* 2003; Shworak *et al.* 2003). Knockout of HS3ST2 does not result in obvious defects in the behavior, fertility, or lifespan of mutant mice nor at least one class of mechanosensory neurons (TrkC-positive neurons) (Hasegawa and Wang 2008). In contrast, RNA interference (RNAi)-mediated knockdown of Hs3st-B, the sole subgroup 2 member in *Drosophila*, produces neurogenic cell fate defects that are similar to Notch loss of function phenotypes (Kamimura *et al.* 2004). Several HST-3s have temporally and spatially restricted expression patterns in the developing vertebrate brain (Yabe *et al.* 2005; Cadwallader and Yost 2006), indicating functions in the nervous system. In addition, recent work with single-chain, variable-fragment HS-specific antibodies has directly demonstrated the association of 3-*O*-sulfation with the *C. elegans* nervous system (Attreed *et al.* 2012). Despite this, an *in vivo* function for HS 3-*O*-sulfate in the nervous system is not known.

Here we show that *C. elegans* lacking the genes encoding the HS 3-*O*-sulfotransferases *hst-3.1* and *hst-3.2* are viable and fertile. Although the overall patterning of the nervous system is intact in both *hst-3.1* and *hst-3.2* mutants, we have identified a function of HS 3-*O*-sulfation in neurite branching of select neurons. Both the hermaphrodite-specific neuron (HSN) and neurite branches in AIY interneurons dependent on overexpression of the extracellular cell adhesion molecule *kal-1* (which is mutant in patients with Kallmann syndrome/idiopathic hypogonadotropic hypogonadism [IHH]) require HS 3-*O*-sulfation. Intriguingly, *hst-3.1* and *hst-3.2* are both required nonredundantly for HSN development, but only *hst-3.2* is necessary for *kal-1*−dependent branches. Expression studies of HS 3-*O*-sulfotransferases and transgenic rescue data suggest that at least some of the genes can function cell nonautonomously. Taken together, our data are consistent with HS 3-*O*-sulfation playing highly specific roles during neural development.

## Material and Methods

### Strains

All strains were maintained and assayed at 20° with standard techniques (Brenner 1974). Mutant alleles used in this study are as follows: *hst-3.2(tm3006)X*, *hst-3.2(tm3208)X*, *hst-3.2(dz171)X*, *hst-3.2(dz169)X*, *hst-3.2(dz164)X*, *hst-3.1(tm734)II*, *hst-3.1(ok1246)II*, *kal-1(gb503)I*, and *kal-1(ok1056)I*. The alleles *tm3006*, *tm734*, and *dz171* were backcrossed at least four times before analysis.

### Characterization and isolation of mutant alleles

The extent of genomic deletions in *hst-3.2(tm3006)*, *hst-3.2(tm3208)*, and *hst-3.1(tm734)* was determined by PCR amplification and sequencing. For cDNA analyses, total RNA was isolated from N2 wild-type and mutant worms and reverse transcribed using a SuperScript III First-Strand Synthesis System kit (Invitrogen, catalog number: 18080-051). The cDNA of mutants (*tm3006*, *tm3208*, *tm734*) was amplified using gene specific primers and sequenced to determine the consequence of the genomic deletion in different alleles. The extent of the genomic deletion in *hst-3.1(ok1249)* was determined by PCR amplification and sequencing of genomic DNA. The point mutant alleles in *hst-3.2* were recovered in an expanded forward genetic screen for modifiers of the *kal-1*−dependent branching phenotype in AIY neurons (C. A. Diaz-Balzac and H. E. Bülow, unpublished data) as previously described (Bülow *et al.* 2002).

### Molecular biology and transgenesis

To assemble tissue-specific expression constructs, the *hst-3.2* cDNA was amplified from wild-type N2 total cDNA with gene specific primers containing *Kpn*I and *Xba*I restriction sites. The amplified cDNA was cloned under control of the following promoters: hypodermal *dpy-7*, body wall muscle *myo-3*, pan-neuronal *rgef-1(F25B3.3)* and an AIY-specific *ttx-3* promoter. All plasmids contained the unc-54 3′UTR and PCR amplified sections were verified by sequencing. All plasmid sequences are available upon request. For rescue experiments, the tissue specific *hst-3.2* expression constructs were injected into EB118 (*otIs76mgIs18*; *hst-3.2(tm3006*)) at 5 ng/µL together with *myo-3*::*mCherry* and *rol-6(su1006)* as dominant injection markers at 50 ng/µL each. The *hst-3.2* genomic region (fosmid WRM0632CA04) was injected into EB1913 (*zdIs13*; *hst-3.2(dz171*)) at 1.25 ng/µl together with *ttx-3*::*mCherry* and *rol-6(su1006)* as dominant injection markers at 50 ng/µl each.

A *hst-3*.1 transcriptional reporter was constructed by amplifying 2409 nt upstream of the predicted *hst-3.1* start codon, and inserting it into *pPD95.75* (gift of Andy Fire, Stanford University). To generate the *hst-3.2* transcriptional reporter line, a fragment containing an intercistronic sequence that directs SL2 splicing of a yellow fluorescent protein (YFP) under control of *hst-3.2* regulatory elements, was PCR amplified from *pBALU10* and inserted immediately after the *hst-3.2* stop codon in fosmid WRM0632CA04 using a recombineering approach (Tursun *et al.* 2009). The *hst-3.1* and *hst-3.2* reporters were injected into wild-type N2 at 20 ng/µL together with the dominant injection marker *rol-6(su1006)* at 100 ng/µL.

### RNAi-mediated gene knockdown

RNAi by feeding was performed as previously described (Kamath and Ahringer 2003) using *zdIs13* or *otIs76mgIs18* to label HSN motor neurons and AIY interneurons that overexpress *kal-1*, respectively. The *hst-3.2* and *kal-1* RNAi constructs were made by subcloning the open reading frames from *pttx-3*::*hst-3.2* and *pttx-3*::*kal-1* (Bülow *et al.* 2002), respectively, via *Kpn*I/*Xba*I digestion into *pPD129.36* (a gift from Andy Fire).

### Scoring of neuroanatomy

Neuroanatomical defects were scored in L4 or young adults. Worms were anesthetized with 10 mM sodium azide and mounted on 5% agarose pads for analysis on a Zeiss Axioimager Z1 compound microscope. All phenotypes were scored for individual neurons and error bars represent the standard error of proportion.

### Statistical analysis

For all proportions statistical significance was calculated using the z-test whereas averages were compared using the two-tailed Student’s *t*-test, both with the Bonferroni correction where applicable. All error bars represent the standard error of proportion. Statistical significance is indicated throughout the paper as: ns: not significant, *P < 0.05, **P < 0.005, ***P < 0.0005.

## Results

### The *C. elegans* HS 3-*O*-sulfotransferases *hst-3.1* and *hst-3.2* are dispensable for viability

**Figure 2:**
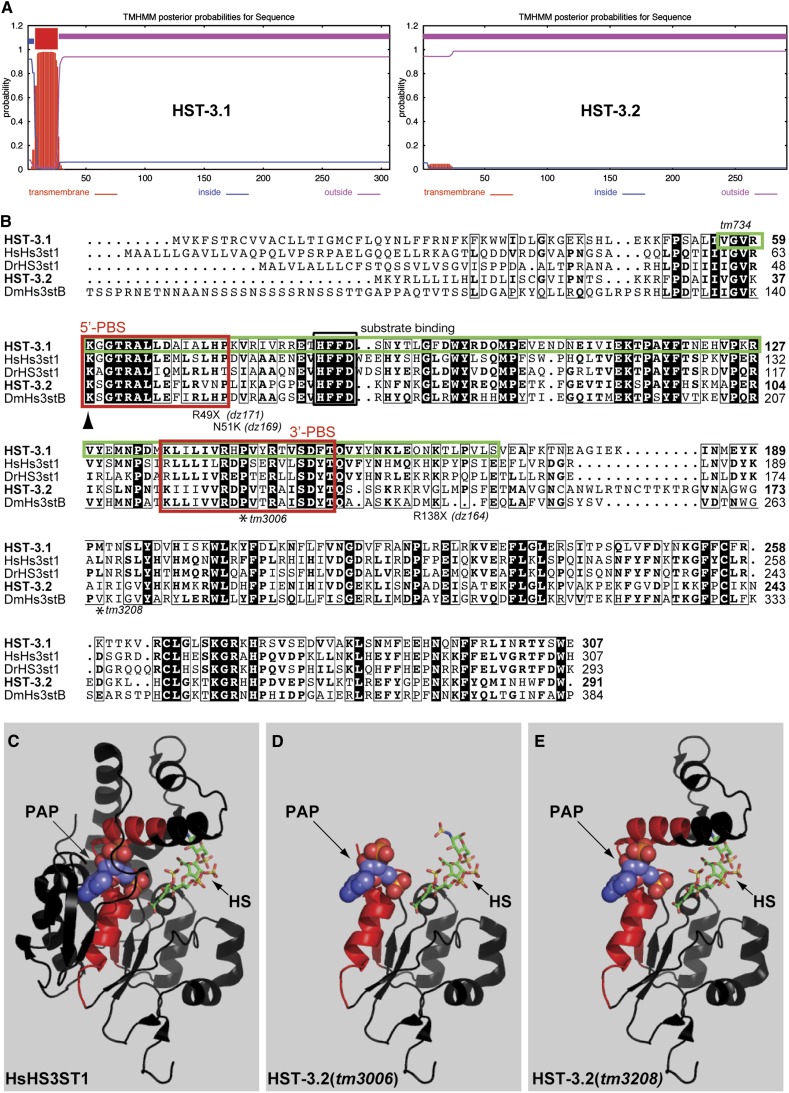
Alignment of HS 3-*O*-sulfotransferases and molecular modeling of deletion mutants. (A) Hydrophathy plots of predicted *C*. *elegans* HS 3-*O*-sulfotransferases HST-3.1 and HST-3.2 using a hidden Markov Model (Krogh *et al.* 2001) at http://www.cbs.dtu.dk/services/TMHMM/. (B) Alignment of predicted *C. elegans* HS 3*-O*-sulfotransferases HST-3.1 and HST-3.2 with human, fish, and drosophila homologs. Similar amino acids are boxed with a thin black line and identical amino acids are shaded in black. Boxed in red are the 3′-PAPS and 5′-PAPS cofactor binding sites, respectively. Boxed in a thick black line is the substrate-binding site. Boxed in green is the predicted deletion in the *hst-3.1(tm734)* allele. The *hst-3.2* point mutant alleles are indicated above or below the sequence and an asterisk marks the predicted last conserved amino acid encoded by the deletion alleles *tm3006* and *tm3208*. Numbers on the right denote amino acid position. (C) Crystal structure of human HsHS3ST1 (PDB:1ZRH). Shown in red are the PAPS binding residues. The cofactor PAPS is shown as a sphere model in blue and the HS substrate as a stick model. (D) Model of the predicted *C. elegans* HST-3.2 protein encoded by the *tm3006* deletion allele. The model was derived utilizing the HsHS3ST1 crystal structure. (E) Model of the predicted *C. elegans* HST-3.2 protein encoded by the *tm3208* deletion allele. The model was derived using the HsHS3ST1 crystal structure.

The *C. elegans* genome encodes two predicted HS 3-*O*-sulfotransferases, HST-3.1 and HST-3.2, that phylogenetically cluster with enzymes of subgroup 1 and 2, respectively (Figure 1B). HST-3.1 is a predicted type II transmembrane Golgi protein of 307 amino acids, whereas HST-3.2 is a predicted secreted protein of 291 amino acids without an obvious transmembrane domain (Figures 1 and 2A). To study the role of HS 3-*O*-sulfation *in vivo*, we obtained deletion alleles for *hst-3.1* and *hst-3.2* (Figure 1, C and D). Based on genomic and cDNA analyses, the *hst-3.1(tm734)* allele produces an in-frame deletion that removes close to half of the sulfotransferase domain, including the two obligate cosubstrate-binding sites (Figure 2, B and E). We conclude that the *tm734* represents a strong, if not complete, loss of function allele. The *hst-3.2(tm3006)* deletion allele (Figure 1) results in a protein that lacks more than two thirds of the sulfotransferase domain, including part of the 3′-PAPS (phosphoadenosyl-phosphosulfate) and the substrate binding sites and is a predicted strong loss of function allele (Figures 1 and 2, B C). A second allele, *hst-3.2(tm3208)*, encodes a protein that leaves a structural determinant of the enzyme partially intact, namely a loop that is predicted to partake in the formation of the groove in which the HS substrate binds (Figures 1 and 2, B and C) (Edavettal *et al.* 2004). In addition, we identified three-point mutant alleles of *hst-3.2* that result in stop codons after 49 amino acids (*dz171*), 138 amino acids (*dz164*), or a N51K missense mutation (*dz169*) (Figures 1 and 2, B and C). Both single and double mutants of *hst-3.1* and *hst-3.2* are viable and fertile and display no obvious morphological defects (Figure 1E).

### Animals with mutations in *hst-3.1* and *hst-3.2* display no major neuroanatomical defects

Primed by previous studies that showed other HS modifications to be required for nervous system development in both *C. elegans* and in vertebrates (Bülow and Hobert 2004; Pratt *et al.* 2006), we sought to determine whether HS 3-*O*-sulfotransferases are also necessary for neural development. Using a panel of fluorescent reporter lines, we found the overall structure of the nervous system, including the placement of the major nerve bundles, separation of the major ganglions and the patterning and axonal guidance of many individual neurons (including interneurons, motor neurons and sensory neurons) intact in *hst-3.1* and *hst-3.2* mutants (Table 1). Moreover, of several cellular contexts tested (DA/DB motor neurons, amphid neurons, and phasmid neurons), only in DA/DB motor neurons did the *hst-3.1*; *hst-3.2* double-mutant display a moderate enhancement compared to the single mutants. The *hst-3.1*; *hst-3.2* double mutant displayed a guidance defect of DA/DB motor neurons in 13% of the animals *vs.* 3% and 2% in the *hst-3.1* or *hst-3.2* single mutants, respectively (*P =* 0.014). These results suggest that *hst-3.1* and *hst-3.2* are individually largely dispensable for axonal guidance and do not serve generally redundant functions.

**Table 1:** Neuroanatomical analyses in HS 3-*O*-sulfotransferase mutants

Neurons Examined (Transgenic Marker Used)	Defects Observed*^a^*	
	*hst-3.1(tm734)*	*hst-3.2(tm3006)*
Sensory neurons		
AFD	–	nd
Amphid neurons (DiI)	14%	9%
Phasmid neurons (DiI)	–	–
AVM neuron (*zdIs5*)		
Ventral axon guidance	–	–
Cell migration	–	–
PVM neuron (*zdIs5*)		
Ventral axon guidance	–	–
Cell migration	–	–
ALM neurons (*zdIs5*)		
Axon termination	–	–
Axon guidance	–	–
PVD neurons (*otIs182*)		
Dendrite branching	–	–
Interneurons		
AIY neurons (*mgIs18*)	–	–
PVT neuron (*otIs39*)	nd	–
PVQ neurons (*hdIs26*)	–	19%
Motor neurons		
D-type neurons (*juIs76*)*^b^*	nd	–
VC neurons *(vsIs13)**^c^*	nd	–
DA/DB neurons (*evIs32b*)*^b^*		
Fasciculation VNC	–	–
Midline L/R choice	–	–
Fasciculation DNC	–	–
Other		
CAN cell migration (*otIs33*)	–	–

a Percent of animals defective is shown if statistically different from controls (N = 25−100). Not different from control is indicated by – and not determined by nd.

b D-type and DA/DB motor neurons were scored for fasciculation defects in the ventral nerve cord (VNC), midline L(eft)/R(ight) commissural guidance choice, circumferential commissural growth, and fasciculation of the dorsal nerve cord (DNC) as described (Bülow and Hobert 2004).

c VC neurons were scored for total number of neurons, fasciculation in the VNC, and of VC4 and VC5 neurons at the vulva.

### *hst-3.1* and *hst-3.2* are required in context-dependent combinations for neurite branching

**Figure 3:**
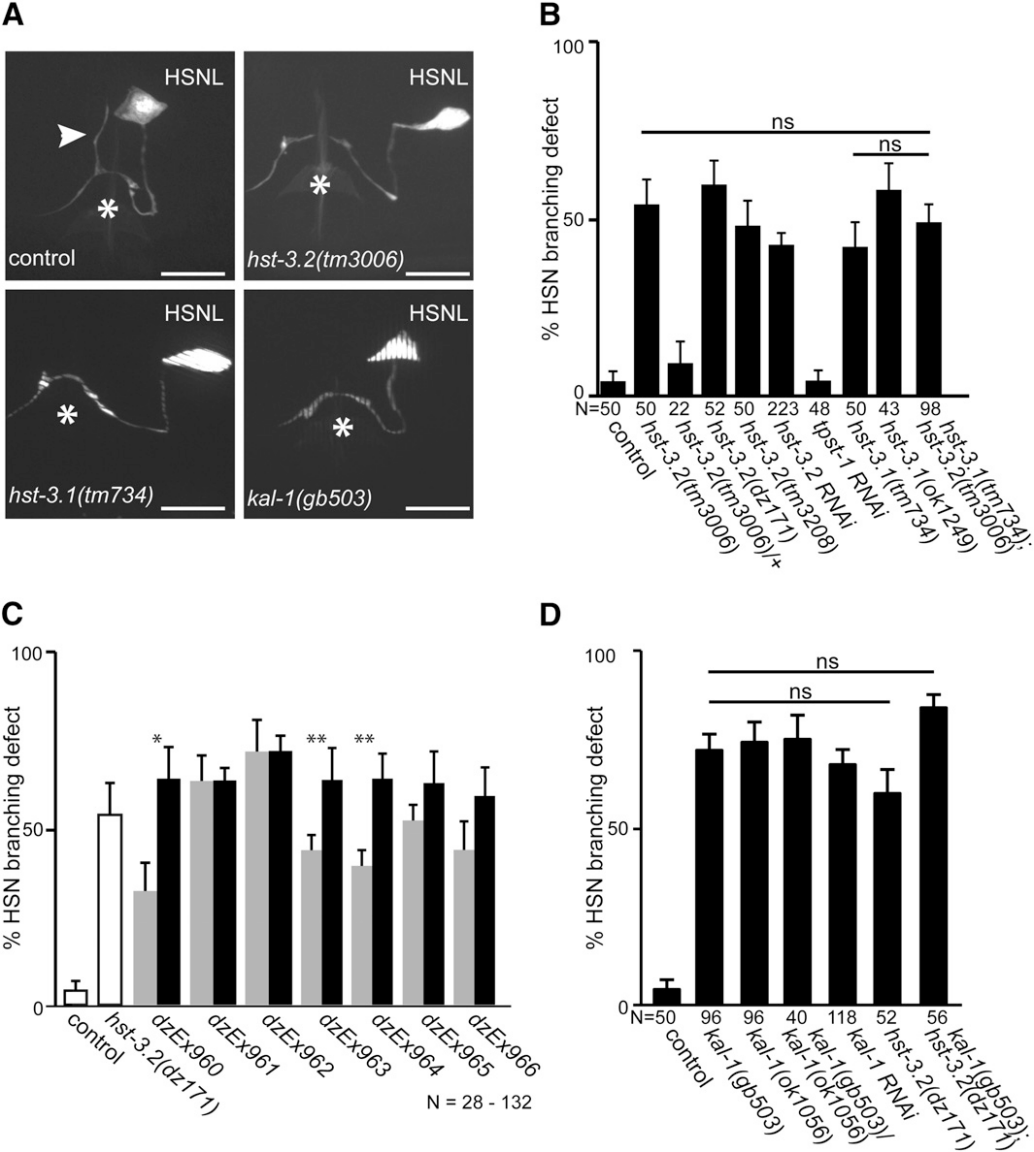
Axonal branching defects of HSN in *hst-3.1*, *hst-3.2* and *kal-1* mutants. (A) Schematic of HSN’s axonal branch. In wild-type animals, a small branch (indicated by an arrowhead) emanating from the HSN axon can been seen at the vulva (indicated by an asterisk). In *hst-3.2*, *hst-3.1*, and *kal-1* mutant animals, this branch is absent. Anterior is to the left in all panels. Scale bars indicate 10 µm. (B) Quantification of the absence of HSN’s axonal branch for genotypes indicated. (C) Quantification of *hst-3.2* genomic rescue of the HSN branch phenotype. Transgenic animals were created in a *hst-3.2 (dz171)* mutant background by injecting the *hst-3.2* containing fosmid WRM0617bB10 (1.25 ng/μL) with *ttx-3*::*mCherry* and *rol-6(su1006)* as dominant injection markers at 50 ng/µL each. Data for several transgenic lines are shown (gray bars) together with isogenic nontransgenic siblings of the same lines (black bars). (D) Genetic interaction of *hst-3.2* and *kal-1*. Data for *hst-3.2(dz171)* is identical to data in B and shown for comparison only.

Because we did not identify general neuroanatomical defects, we investigated more specific aspects of the nervous system and identified defects in the HSN. HSNs are born embryonically in the tail and migrate anteriorly to their final position slightly posterior to the vulva during larval stages (Desai *et al.* 1988). By the L3 larval stage, an axon is sent ventrally from the cell body into the ventral nerve cord, where it fasciculates with other neurons and projects anteriorly to the nerve ring (Desai *et al.* 1988), the major neuropil in the worm. Near the vulva, a small branch emanates dorsally from the HSN axon (Figure 3A) (Garriga *et al.* 1993). Many synapses between HSN and the egg-laying muscle vm2 and the VC4 and VC5 neurons are found within and around this branch (White *et al.* 1986; Garriga *et al.* 1993). HSN cell body migration and HSN axon guidance are not affected by loss of either *hst-3.1* or *hst-3.2* (data not shown). However, genetic removal of *hst-3.1* or *hst-3.2* results in defects in HSN branch formation at the vulva (Figure 3B) without obvious defects in egg laying rate compared to control animals (data not shown). Similar branching defects are seen in other alleles of *hst-3.1* and *hst-3.2* (including both deletion and point mutant alleles) and upon RNAi-mediated gene knockdown of *hst-3.2* but not an unrelated sulfotransferase (*tpst-1*, tyrosine sulfotransferase) in wild-type worms (Figure 3B). Finally, the branching defect was partially rescued by injection of DNA containing the *hst-3.2* genomic locus (Figure 3C). A double mutant of *hst-3.1* and *hst-3.2* did not show enhancement of the phenotype when compared with either of the single mutants (Figure 3B), which indicated that both HS 3-*O*-sulfotransferases are acting within the same genetic pathway and serve nonredundant functions to regulate HSN axonal branching.

**Figure 4:**
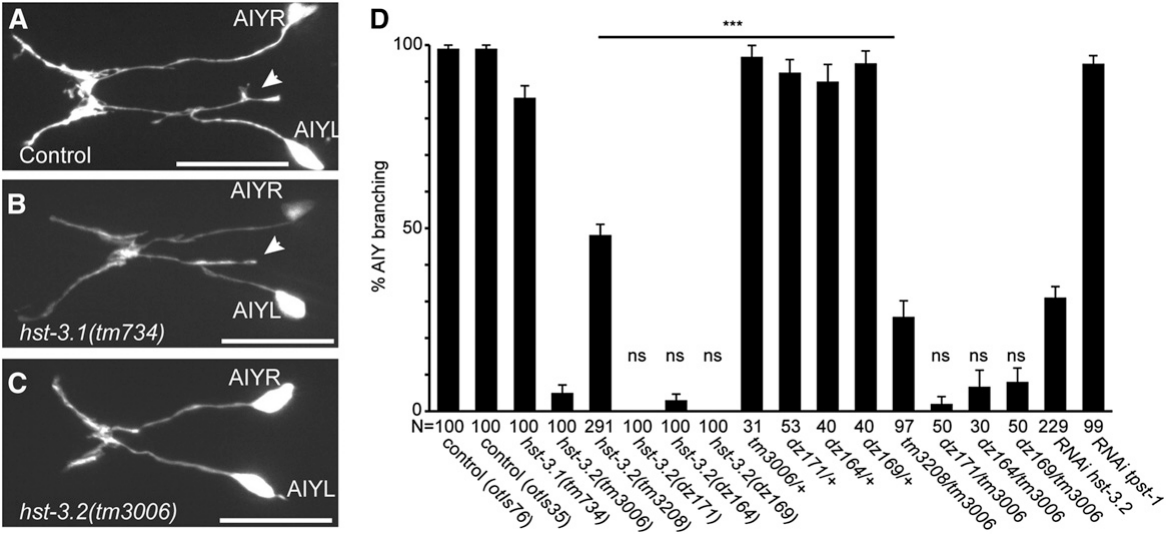
Suppression of *kal-1*−dependent branching in HS 3-*O*-sulftransferase mutants in AIY interneurons. (A−C) Epifluorescent micrographs showing ventral views of *kal-1*−dependent branches in AIY interneurons in controls (A), *hst-3.2* mutant (B), and *hst-3.1* (C) mutant animals. Anterior is to the left in all panels. A white arrowhead indicates *kal-1*−dependent branches, and scale bars = 20 µm. (D) Quantification of *kal-1*−dependent branching with percent branching shown ± the SE of proportion. Statistical significance is indicated by asterisks. Mutant genotypes were assayed in homozygous, heterozygous, or transheterozygous combinations of *otIs35* or *otIs76 kal-1*-misexpressing transgenes, which we previously showed to be off statistically indistinguishable penetrance (Bülow *et al.* 2002). Note that we did observe the molting defects that have been described for RNAi against *tpst-1* (Kim *et al.* 2005), suggesting effective knockdown of *tpst-1*.

Because we had previously shown that other HS modifications are important for neurite branching in *C. elegans* (Bülow *et al.* 2002), we tested whether neurite branching in AIY interneurons as a result of overexpression of *kal-1* (Figure 4A) (Bülow *et al.* 2002) is dependent on HS 3-*O*-sulfotransferases. *C. elegans kal-1* encodes the homolog of KAL1/anosmin-1, an extracellular matrix protein, which is mutant in patients with the X-linked form of Kallmann syndrome (for review, see Hardelin and Dodé 2008). The AIY branching phenotype is dependent on the presence of several HS modification enzymes including the C5 glucuronyl epimerase, *hse-5*, and the HS 6-*O*-sulfotransferase, *hst-6* but is less dependent on the HS 2-*O*-sulfotransferase, *hst-2* (Bülow *et al.* 2002; Bülow and Hobert 2004). We found that *kal-1*−dependent branches in AIY require *hst-3.2* but not *hst-3.1* (Figure 4, B and C). All *hst-3.2* alleles behaved recessively and failed to complement each other (Figure 4D). Moreover, suppression of *kal-1*−dependent axonal branching in AIY was phenocopied by RNAi-mediated gene knockdown of *hst-3.2* but not an unrelated sulfotransferase (*tpst-1*, tyrosine sulfotransferase) in wild-type animals (Figure 4D). The phenotype of the *tm3208* deletion allele is less severe than the remaining alleles and more severe when placed *in trans* to the deletion allele *tm3006* (Figure 4D). Thus, *tm3208* presents a hypomorphic allele of *hst-3.2* that retains some function, whereas the remaining alleles of *hst-3.2* may be strong if not complete loss of function alleles.

To further investigate the genetic relationship between *kal-1* and HS 3-*O*-sulfotransferases, we first characterized *kal-1* loss of function mutants. In *C. elegans*, loss of *kal-1* has been shown to result in defects in epidermal attachment (Rugarli *et al.* 2002) and delays in ventral epidermal closure (Hudson *et al.* 2006). Moreover, some extra branches in RIC and the male specific interneuron EF3 have been documented (Rugarli *et al.* 2002), but no systematic neuroanatomical study of *kal-1* loss of function mutants has been undertaken. We found that *kal-1* mutant animals do not display obvious defects in the overall organization of the nervous systems, nor in a diverse panel of individual neurons and neuronal classes (Table 2). However, we identified a branching defect in HSN neurons similar to the abnormalities observed in *hst-3.1* and *hst-3.2* mutants. A total of 72% or 74% of animals carrying either the *gb503*- or *ok1056*-predicted null alleles of *kal-1* (Hudson *et al.* 2006), respectively, displayed a loss of HSN synaptic branches (Figure 3D). Moreover, *gb503* and *ok1056* failed to complement each other and the HSN phenotype was observed by RNAi mediated gene knock down of *kal-1* in wild-type animals (Figure 3D). Similar to *hst-3.1* and *hst-3.2*, *kal-1* mutants do not show a defect in egg laying rate compared with wild-type control animals (data not shown). Thus, like loss of *hst-3.1* and *hst-3.2*, *kal-1* mutants do not display major defects in neuronal patterning but do exhibit defects in the formation of the HSN synaptic branch (Table 2, Figure 3). The defect in HSN branching in *kal-1* mutants is statistically indistinguishable from *hst-3.2* mutants. To determine whether *hst-3.2* acts in the *kal-1* pathway or also serves functions that are independent of *kal-1*, we constructed a *kal-1*; *hst-3.2* double mutant. We found the HSN defect in the *kal-1*; *hst-3.2* double mutant not significantly enhanced compared to the *kal-1* single mutant (Figure 3D), indicating that *hst-3.2* and *kal-1* act within the same genetic pathway to promote neurite branching in HSN neurons.

**Table 2:** Neuroanatomical analyses *kal-1* mutants

Neurons Examined (Transgenic Marker Used)	Defects Observed*a* *kal-1(gb503)*
Sensory neurons	
AFD	nd
Amphid neurons (DiI)	–
AVM neuron (*zdIs5*)	
ventral axon guidance	–
cell migration	
PVM neuron (*zdIs5*)	
ventral axon guidance	–
cell migration	
ALM neurons (*zdIs5*)	
axon termination	–
axon guidance	–
Interneurons	
AIY neurons (*mgIs18*)	–
Motor neurons	
D-type neurons (*juIs76*)*^b^*	–
VC neurons *(vsIs13)*	nd
DA/DB neurons (*evIs32b*)	
fasciculation DNC	–
Other	
CAN cell migration (*otIs33*)	–

a Percent of animals defective is shown if statistically different from controls (N = 25-100). No defect is indicated by – and not determined by nd.

b D-type and DA/DB motor neurons were scored for fasciculation defects in the ventral nerve cord (VNC), midline L(eft)/R(ight) commissural guidance choice, circumferential commissural growth, and fasciculation of the dorsal nerve cord (DNC) as described (Bülow and Hobert 2004).

### The HS 3-*O*-sulfotransferase *hst-3.2* can function nonautonomously to mediate *kal-1* function *in vivo*

**Figure 5:**
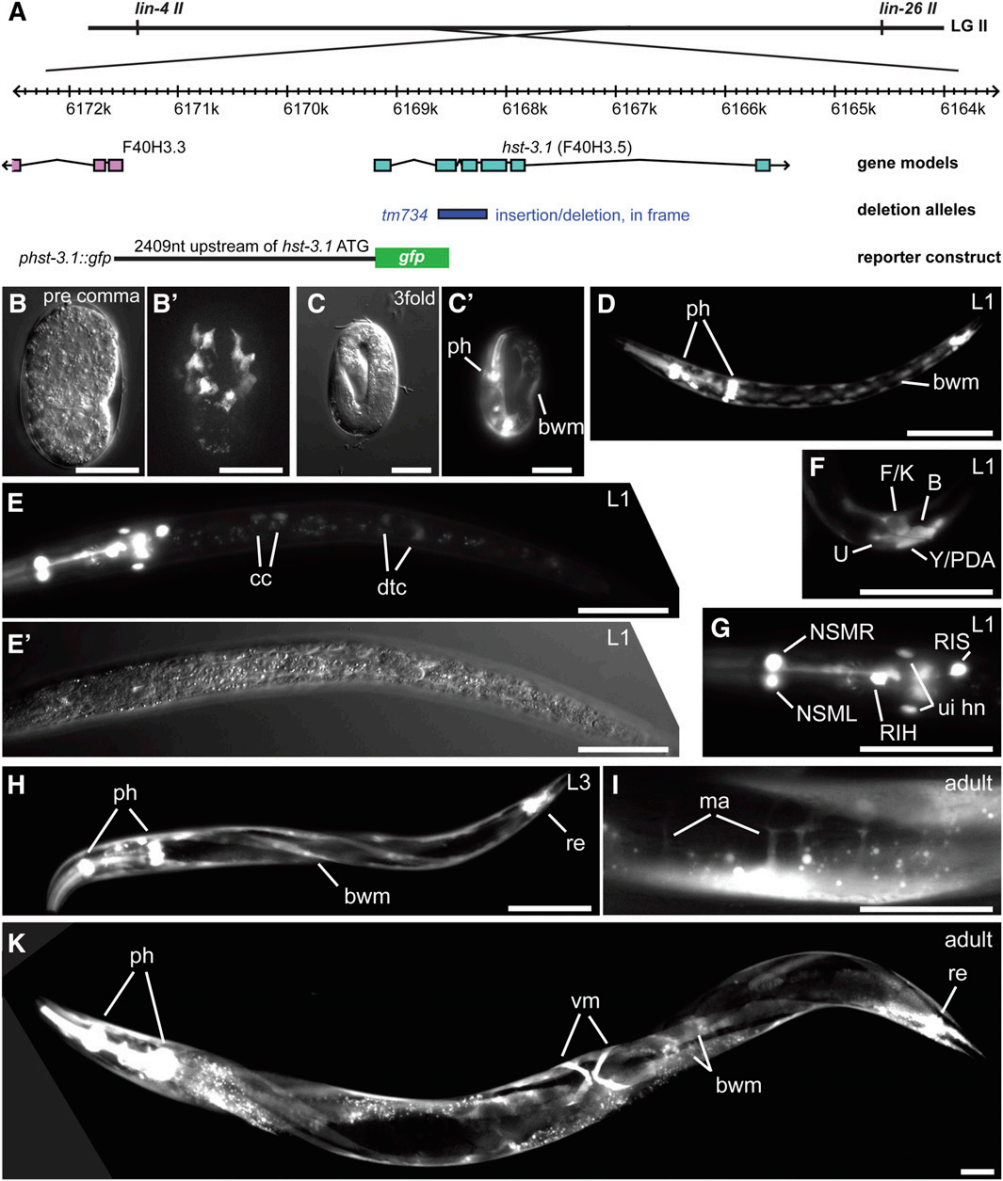
The HS 3-*O*-sulfotransferases 1 is expressed in select neurons and primarily mesodermal tissues. (A) Schematic of a transcriptional reporter for *hst-3.1* (F40H3.5). LG II (linkage group) on which *hst-3.1* is located is shown. The genomic structure of *hst-3.1* is shown with exons in blue boxes and introns represented by black lines with the extent of the *tm734* deletion indicated below. The *hst-3.1* transcriptional reporter was generated by PCR fusion of 2409 nt upstream of the *hst-3.1* start codon to GFP. Straight arrows indicate the direction of transcription of partially shown genes. (B–J) The *hst-3.1* reporter shows expression from embryonic stages through larval stages in the pharynx (ph) and body wall muscles (bwm). Neuronal expression is restricted to NSM, RIS, RIH, and an unidentified pair of head neurons (ui hn). Additional expression is observed in the coelomocytes (cc), the distal tip cell (dtc), and rectal cells (U,F/K, B AND Y/PDA). In adults, expression is predominantly seen in body wall muscles, vulva muscles (vm), the muscle arms (ma), the pharynx, and the rectal epithelium (re). Scale bars = 20 µm.

**Figure 6:**
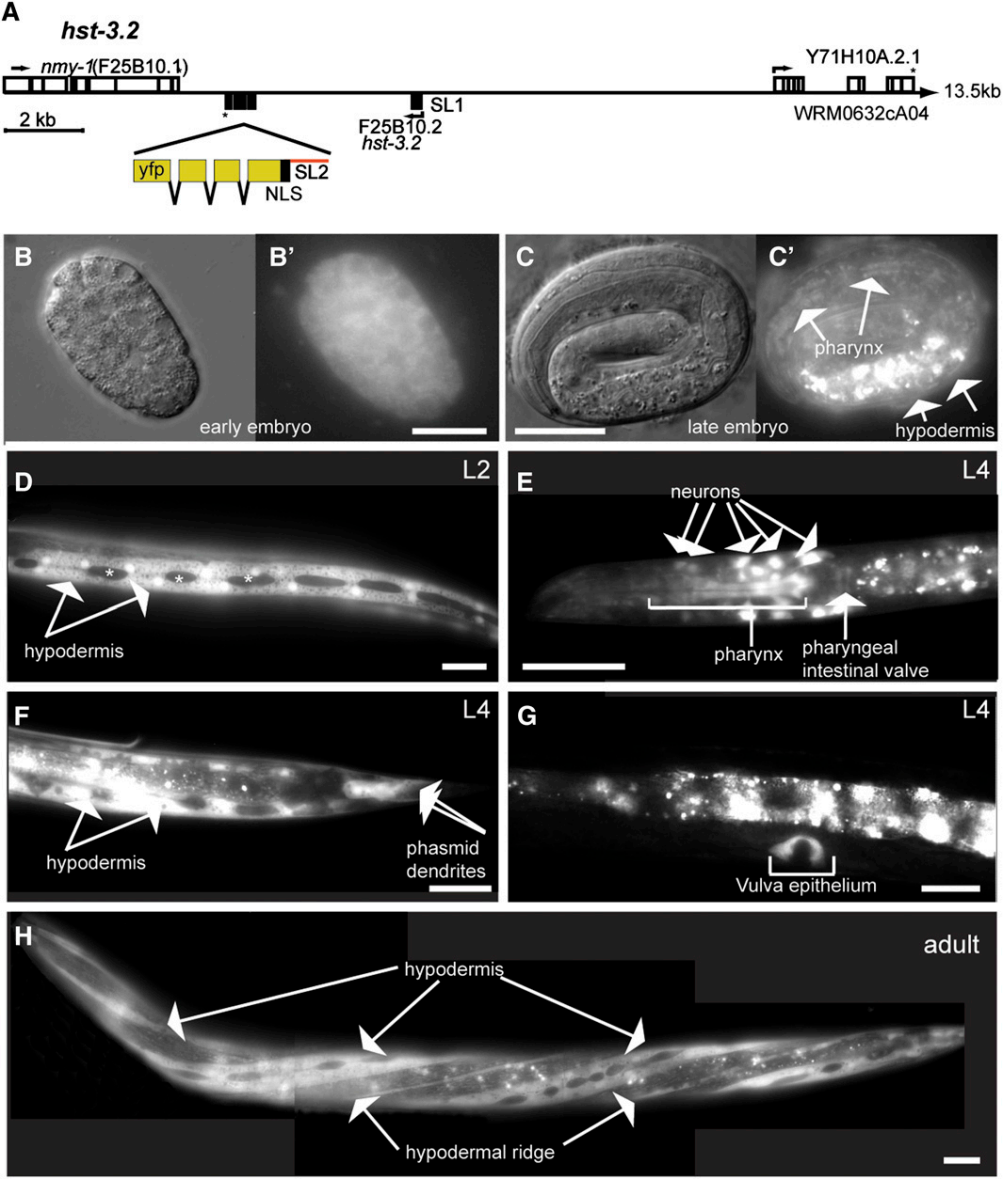
The HS 3-*O*-sulfotransferases 2 is expressed in ectodermal tissues. (A) Schematic of a transcriptional reporter for *hst-3.2*. Shown are 20 kb around the *hst-3.2* locus. YFP with a nuclear localization signal (NLS) under control of an intercistronic region directing SL2-splicing (red) is inserted after the stop codon for *hst-3.2* in fosmid WRM063bF12. Black boxes denote coding exons of *hst-3.2*, and white boxes denote coding exons of surrounding genes. Predicted start codons and direction of transcription are indicated by a rectangular arrow and stop codons by an asterisk. Straight arrows indicate the direction of transcription of partially shown genes. Extent of fosmid clone in kilobases (kb) is indicated on the right. Gene names are as shown (www.wormbase.org; version WB213). SL2: splice leader 2 (B−H). The *hst-3.2* reporter shows widespread expression from embryonic stages through larval stages in hypodermal and about two dozen neurons in the head and tail. In adults, expression is predominantly seen in hypodermal tissues with the seam cells visibly excluded. Scale bars indicate 20 µm.

To define whether HS 3*-O*-sulfotransferases act more likely autonomously, *i.e.*, in the affected neurons or nonautonomously, *i.e.*, in surrounding tissues to control neurite branching we first determined where *hst-3.1* and *hst-3.2* are expressed. We constructed the *hst-3.1* transcriptional reporter by polymerase chain reaction (PCR) fusion of 2409 nt upstream of the *hst-3.1* start codon directly to GFP (Hobert 2002) (Figure 5A). For the *hst-3.2* reporter, we engineered a YFP after the stop codon of the gene in the context of the whole genomic sequence of *hst-3.2* (Tursun *et al.* 2009) (Figure 6A). This construct should contain most, if not all regulatory elements driving expression of *hst-3.2*. We then generated transgenic animals carrying reporters for each gene.

A *hst-3.1* reporter displayed limited expression during early embryogenesis, but was visible in the pharynx and bodywall muscle by the embryonic threefold stage. During larval and adult stages, the reporter continued to be expressed in body wall muscle, vulval muscle and the pharynx. In addition, we detected expression in at least six neurons in the head, including the NSM, RIH, RIS, and an unidentified pair of neurons as well as some select epithelial cells (Figure 5). No obvious expression was seen in either AIY or HSN interneurons. In contrast, the *hst-3.2* reporter displayed almost ubiquitous expression early during embryogenesis but, during larval stages and adulthood became more restricted to epithelia and neurons (Figure 6). We detected expression in the hypodermis (excluding obvious expression in the seam cells) and the vulval epithelium. In addition, we observed expression in about two dozen head neurons and possibly the enteric muscle (Figure 6). Again, no obvious expression was seen in HSN neurons. We suggest that the two sulfotransferases *hst-3.1* and *hst-3.2* are (1) expressed in largely complementary patterns and (2) may serve nonautonomous functions, at least in the cellular contexts of AIY and HSN neurons.

**Figure 7:**
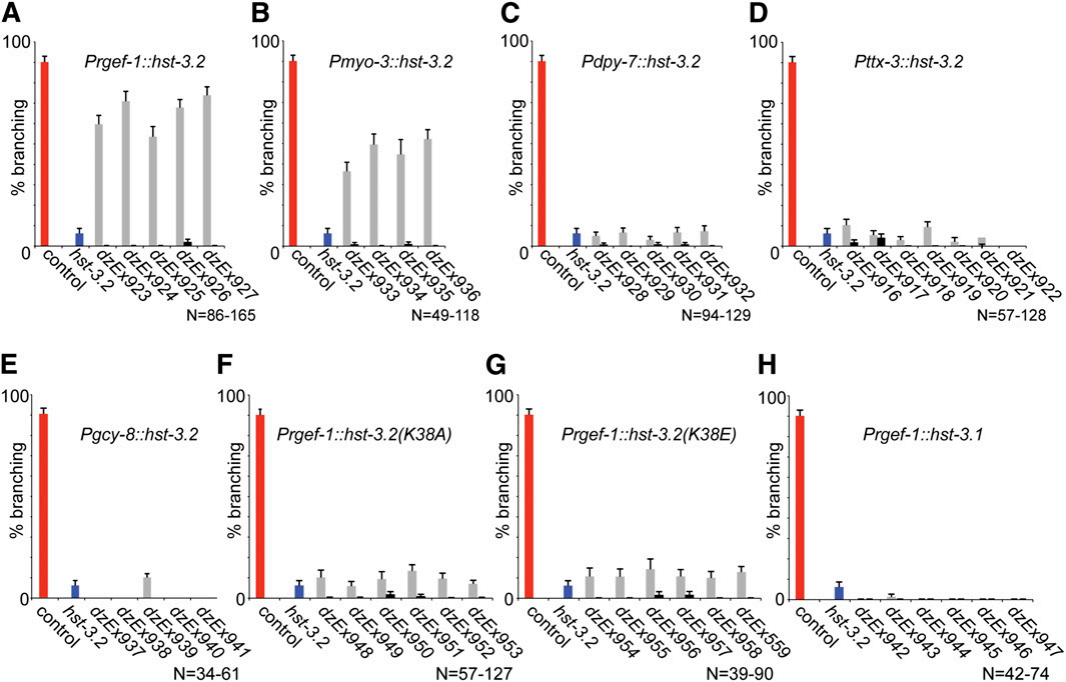
Enzymatic activity of HST-3.2 is required nonautonomously for *kal-1*−dependent branching. (A–H) Rescue of *kal-1*−dependent branching in AIY neurons in *hst-3.2* mutant animals. Transgenic animals were created in a *hst-3.2(tm3006)* mutant background by injecting the respective cDNAs under control of a pan-neuronal (*rgef-1*), muscle specific (*myo-3*), hypodermal specific (*dpy-7*), AIY specific (*ttx-3*) or AFD specific (*gcy-8*) promoters at 5 ng/µL together with *myo-3*::*mCherry* and *rol-6(su1006)* as dominant injection markers at 50 ng/µL each. Data for several transgenic lines are shown (gray bars) together with isogenic nontransgenic siblings of the same lines (black bars). Values for controls (red bars) and *hst-3.2* mutant (blue bar) are identical in all panels and shown for comparison only.

To corroborate these findings, we focused on the *kal-1*−dependent branching phenotype in AIY interneurons and performed cell-specific rescue experiments. Transgenic expression of *hst-3.2* in all neurons or body wall muscle rescues loss of *hst-3.2*, *i.e.*, reverses the suppression of branching (Figure 7, A and B). In contrast, expression of *hst-3.2* in surrounding hypodermal tissues, AIY interneurons or AFD sensory neuron (a presynaptic partner to AIY) fails to reverse the suppression of branching (Figure 7, C−E). To determine whether enzymatic activity of *HST-3.2* is required for *kal-1−*dependent branches, we tested two pan-neuronally expressed *hst-3.2* point mutants of lysine 38. Lysine 38 in *C. elegans* HST-3.2 is conserved in all HS 3-*O*-sulfotransferases (Figure 2B). Mutating the analogous lysine in the vertebrate HS 3-*O*-sulfotransferase-1 abolishes enzymatic activity without affecting protein stability (Edavettal *et al.* 2004). We found that expressing catalytically dead K38 versions of *hst-3.2* fails to rescue loss of *hst-3*.2, *i.e.*, reverse suppression of branching (Figure 7, F and G). Similarly, pan-neuronal expression of the HS 3-*O*-sulfotransferase *hst-3.1* does not reverse the suppression of branching (Figure 7H). These findings indicate that enzymatic activity of HST-3.2 in neurons or muscle is sufficient to nonautonomously mediate the *kal-1*−dependent branching phenotype in AIY neurons. Moreover, unlike in HSN neurons *kal-1* mediated neurite branching in AIY neurons requires a HS motif that comprises 3-*O*-sulfation introduced by the subgroup two HS 3-*O*-sulfotransferase HST-3.2, rather than the subgroup one HS 3-*O*-sulfotransferase HST-3.1.

## Discussion

We describe here the first genetic and molecular analysis of animals predicted to lack all HS 3-*O*-sulfotransferase activity. Single and double mutants appear surprisingly normal, both morphologically and neuro-anatomically, demonstrating that HS 3-*O*-sulfation is not essential for overall development of *C. elegans* and does not play a major role in the patterning of the *C. elegans* nervous system. However, we identified a role for HS 3-*O*-sulfation in neurite branching in select neurons and provide genetic evidence that distinct HS 3-*O*-sulfation patterns mediate neurite branching in a context-dependent manner.

### Kallmann syndrome and HS sulfation

Previous reports have shown that *kal-1* in *C. elegans* is required for the proper formation of the male tail and can act as a regulator of neurite branching in some cellular contexts (Rugarli *et al.* 2002). However, no extensive cell specific analysis in the *kal-1* loss of function mutant has been published to date. The results presented here show that *kal-1* is largely dispensable for the overall formation of the nervous system (Table 2). However, we show that *kal-1* has a branch promoting activity. First, upon loss of *kal-1* function, HSN neurons fail to form axonal branches at the vulva (Figure 3, A and D). Second, overexpression of *kal-1* in AIY neurons results in ectopic branches (Bülow *et al.* 2002). The formation of *kal-1*−dependent branches in AIY interneurons is contingent on several HS modification enzymes, including the HS glucuronyl-epimerase *hse-5* and the HS 6-*O*-sulfotransferase *hst-6* (Bülow and Hobert 2004). We find that these branches are dependent on *hst-3.2* but not *hst-3.1* (Figure 4).

Interestingly, *hst-3.2* function can be provided nonautonomously by muscle and/or neurons but not through expression in the hypodermis, AFD, or AIY interneurons (Figure 7). These data suggest that the HS could be carried by a proteoglycan that is expressed by both muscle and neurons (but not the hypodermis) or, alternatively two different proteoglycans expressed individually in each tissue. Whichever tissue is the source of the 3-*O*-sulfated HS, the interaction of *KAL-1* with this proteoglycan(s), via the HS motif generated by *hst-3.2*, may mediate local cellular adhesion and result in the formation of branches. Taken together, our genetic data are consistent with the hypothesis that KAL-1 could be a protein ligand that requires specifically 3-*O*-sulfated HS to function during development and establish specific neuronal connections in an HS-dependent manner. Because loss of function mutations in a human ortholog of the HS 6-*O*-sulfotransferase *hst-6* have been identified in patients with Kallmann syndrome/IHH (Tornberg *et al.* 2011), the human orthologs of HS 3-*O*-sulfotransferases also represent plausible candidate genes to be mutated in patients with Kallmann syndrome/IHH.

### 3-*O*-sulfated HS epitopes refine the HS landscape to direct neural circuit assembly

Several HS modification enzymes (other than HS 3-*O*-sulfotransferases) have been shown to be required in different combinations for multiple steps of neuronal patterning of both *C. elegans* and vertebrate neurons. For example, retinal ganglion cells in mice require *Hs2st* and *Hs6st1* (encoding the HS 2-*O*-sulfotransferase and a 6-*O*-sulfotransferase, respectively) for different aspects of axonal patterning at the optic chiasma (Pratt *et al.* 2006). In *C. elegans*, axonal patterning of PVQ neurons in the ventral nerve cord requires HS modification enzymes encoded by *hst-2*, *hst-6*, and *hse-5*. In contrast, HSN cell body migration requires the activity of *hst-2* but neither *hst-6* nor *hse-5* (Bülow and Hobert 2004). These studies have lead to the HS code hypothesis which posits that HS modifications modulate ligand and receptor interactions in a cell or tissue specific manner (Holt and Dickson 2005). However, the role of 3-*O*-sulfation in the HS code hypothesis and in neuronal development has not been addressed. Surprisingly, we did not find any obvious developmental defects in mutants of HS 3-*O*-sulfotransferases comparable to mutants in other HS modification enzymes (Table 1). Instead, we found more specific aspects of neuronal development affected such as the formation of select neurite branches: HSN’s axonal branches at the vulva (Figure 3, A and B) and *kal-1*−dependent branches in AIY (Figure 4) (Bülow *et al.* 2002). Interestingly, neither mutations in *hst-3.1* or *hst-3.2* affected the extensive branching of PVD mechanosensory neurons (Halevi *et al.* 2002; Tsalik *et al.* 2003) (Table 1). Taken together, these findings indicate that in HS 3-*O*-sulfotransferases mutants, normal patterning of the HS code is largely intact and that HS 3-*O*-sulfation may serve more specific rather than general functions in branch formation.

If the HS code is largely intact in the 3-*O*-sulfotransferase mutants, then what, if anything, is missing to explain the very specific defects observed in these animals? 3-*O*-sulfotranferases are believed to be the last enzymes to act in the HS modification pathway (Lindahl and Li 2009). This assertion is supported by the fact that some 3-*O*-sulfotranferases localize to the trans Golgi, while other HS modification enzymes are found in the cis and medial Golgi (Pinhal *et al.* 2001; Yabe *et al.* 2001; Nagai *et al.* 2004; Presto *et al.* 2008). In addition, 3-*O*-sulfotranferases have been shown to require highly modified HS as an enzymatic substrate *in vitro* (Liu *et al.* 1999). Taken together, this indicates that the HS code in HS 3-*O*-sulfotransferase mutants may be generally laid out with the exception of local and highly specific modification patterns comprising 3-*O*-sulfated HS that serve to refine the HS code.

HS modification patterns with 3-*O*-sulfation have been suggested to act as protein-binding sites (Lindahl and Li 2009). Specifically, *in vitro* assays have identified three proteins that require 3-*O*-sulfation pattern for high-affinity binding, namely antithrombin (Atha *et al.* 1984), the Herpes simplex viral envelope protein gD (Shukla *et al.* 1999), and cyclophilin B (Vanpouille *et al.* 2007). However, no proteins with developmental functions that bind 3-*O*-sulfated HS have been described. We provide here genetic evidence that the extracellular cell adhesion molecule KAL-1 requires 3-*O*-sulfated HS *in vivo*. Moreover, we find that *kal-1* mediated axonal branching in HSN neurons requires HS with both *hst-3.1*- and *hst-3.2*-derived motifs whereas *kal-1*−dependent axonal branching in AIY requires HS motifs modified by *hst-3.2* but not *hst-3.1*. HS 3-*O*-sulfotranferases can be classified into two subgroups based on *in vitro* substrate specificity (Liu *et al.* 1999) or protein sequence homology (Figure 1B). Our findings thus provide genetic evidence for distinct 3-*O*-sulfated HS modification patterns regulating *kal-1* function in different cellular contexts and suggest an additional layer of complexity in the interaction of proteins with 3-*O*-sulfated HS.

The findings presented here suggest that 3-*O*-sulfation serves to diversify the HS code. In this context, it is interesting to consider the expansion of the gene family that encodes HS 3-*O*-sulfotransferases. The cnidarian *Nematostella* contains 3-*O*-sulfated HS disaccharides, yet the *Nematostella* genome encodes but a single predicted HS 3-*O*-sulfotransferase (Feta *et al.* 2009). The first direct evidence for two types of HS 3-*O*-sulfotransferases is found in mollusks (Gesteira *et al.* 2011). Arthropods (Kamimura *et al.* 2004) and nematodes (this study) also contain both types of HS 3-*O*-sulfotransferases, but the gene families are further expanded to at least seven HS 3-*O*-sulfotranferases of different types in vertebrates (Lindahl and Li 2009). In contrast to cnidarians that have a diffuse nerve net (reviewed in Galliot and Quiquand 2011), mollusks contain a central nervous system with distinct ganglia (Arendt *et al.* 2008). Thus, the expansion of HS 3-*O*-sulfotransferase genes occurs concomitantly with the transition of diffuse nerve nets to more complex nervous systems. Intriguingly, the seven HS 3-*O*-sulfotransferases in vertebrates are expressed in complex overlapping and nonoverlapping expression patterns in the nervous system (Yabe *et al.* 2005; Cadwallader and Yost 2006). We speculate that the combinatorial use of 3-*O*-sulfation of HS is an important contributor to the establishment of a complex nervous system in higher organisms by generating specific high affinity and spatially restricted HS modification patterns that control the complex molecular interplay during development of the neuronal circuitry.
